# Nicotinamide riboside alleviates cisplatin-induced peripheral neuropathy via SIRT2 activation

**DOI:** 10.1093/noajnl/vdac101

**Published:** 2022-06-27

**Authors:** Scarlett Acklin, Ratan Sadhukhan, Wuying Du, Mousumi Patra, Ravi Cholia, Fen Xia

**Affiliations:** Department of Radiation Oncology, University of Arkansas for Medical Sciences, Little Rock, Arkansas, USA; Department of Radiation Oncology, University of Arkansas for Medical Sciences, Little Rock, Arkansas, USA; Department of Radiation Oncology, University of Arkansas for Medical Sciences, Little Rock, Arkansas, USA; Department of Radiation Oncology, University of Arkansas for Medical Sciences, Little Rock, Arkansas, USA; Department of Radiation Oncology, University of Arkansas for Medical Sciences, Little Rock, Arkansas, USA; Department of Radiation Oncology, University of Arkansas for Medical Sciences, Little Rock, Arkansas, USA

**Keywords:** cisplatin, neuropathy, nicotinamide riboside, SIRT2

## Abstract

**Background:**

Chemotherapy-induced peripheral neuropathy represents a major impairment to the quality of life of cancer patients and is one of the most common dose-limiting adverse effects of cancer treatment. Despite its prevalence, no effective treatment or prevention strategy exists. We have previously provided genetic evidence that the NAD^+^-dependent deacetylase, SIRT2, protects against cisplatin-induced peripheral neuronal cell death and neuropathy by enhancing nucleotide excision repair. In this study, we aimed to examine whether pharmacologic activation of SIRT2 would provide effective prevention and treatment of cisplatin-induced peripheral neuropathy (CIPN) without compromising tumor cell cytotoxic response to cisplatin.

**Methods:**

Using von Frey and dynamic hot plate tests, we studied the use of nicotinamide riboside (NR) to prevent and treat CIPN in a mouse model. We also performed cell survival assays to investigate the effect of NAD^+^ supplementation on cisplatin toxicity in neuronal and cancer cells. Lewis lung carcinoma model was utilized to examine the effect of NR treatment on in vivo cisplatin tumor control.

**Results:**

We show that NR, an NAD^+^ precursor and pharmacologic activator of SIRT2, effectively prevents and alleviates CIPN in mice. We present in vitro and in vivo genetic evidence to illustrate the specific dependence on SIRT2 of NR-mediated CIPN mitigation. Importantly, we demonstrate that NAD^+^ mediates SIRT2-dependent neuroprotection without inhibiting cisplatin cytotoxic activity against cancer cells. NAD^+^ may, in fact, further sensitize certain cancer cell types to cisplatin.

**Conclusions:**

Together, our results identify SIRT2-targeted activity of NR as a potential therapy to alleviate CIPN, the debilitating and potentially permanent toxicity.

**Key Points**

- NR supplementation effectively prevents and treats CIPN in a SIRT2-dependent manner.
- NAD^+^ protects neuronal cells without affecting cisplatin cytotoxicity in cancer cells and carcinoma model.

**Importance of the Study**

This is the first study demonstrating that nicotinamide riboside can prevent and treat cisplatin-induced neurotoxicity through SIRT2 activation. It provides a feasible pharmacologic agent to alleviate CIPN which could be evaluated in a clinical trial.

Chemotherapy-induced peripheral neuropathy, a well-known dose-limiting toxicity of chemotherapeutics, poses a significant barrier to the potentially life-saving effects of systemic chemotherapy.^1,2^ The systemic neuronal toxicity can develop from high dose or after cumulative exposure and is closely associated with the narrow therapeutic index of many systemic cancer treatments. Antineoplastic agents, such as platinum-based chemotherapy, are known to induce diffuse, bilateral degenerative changes in peripheral sensation and subsequent alteration in the perception of touch, temperature, and pain. Cisplatin-induced peripheral neuropathy (CIPN) presents clinically as paresthesias or burning, shooting, or electric-shock-like pain, resulting in significant, and often permanent, impediment to the quality of life of patients with cancer.^1,3^ As the prevalence of cancer continues to increase, so does the use of chemotherapy, and the urgency of CIPN continues to increase as no effective treatments or preventive strategies are available.^1,2^

Despite decades of research, not enough is understood about the mechanisms underlying CIPN to effectively prevent and treat the toxicity. Many working models have been proposed, however, the exact pathophysiology remains to be elucidated. While peripheral neurons are known to play an integral role, other cells implicated in CIPN development include macrophages, Langerhans cells, and Schwann cells. Inflammation has been shown to induce macrophage accumulation in the dorsal root ganglia (DRG) as well as loss of intraepidermal nerve fibers due to Langerhans cell proliferation. Finally, chemotherapy agents can promote the formation of inclusion bodies and vacuoles in Schwann cells with subsequent cytotoxicity.^4–6^

Cisplatin, a platinum-based chemotherapeutic, remains first-line treatment in a variety of cancers despite dose limitations caused by its toxicity profile. It exerts its antineoplastic effects through the formation of DNA–platinum adducts,^7–9^ which halt replication forks, interrupt replication and transcription, and induce signaling pathways that result in cell cycle arrest or cell death.^3,7–9^ While this process is necessary to kill cancer cells, its off-target effects on normal tissues must be avoided to prevent toxicity to the patient. Previous studies have shown that cisplatin preferentially binds to and crosslinks the DNA of DRG neurons with a high propensity for platinum adduct formation.^9,10^

The ability of DRG neurons to repair this DNA damage is an important determinant of neurotoxicity severity. Nucleotide excision repair (NER) is known as the primary cellular mechanism through which DNA intrastrand crosslinks are resolved.^11,12^ To effectively repair DNA lesions, NER requires the complex coordination of major protein groups^13^ which could serve as potential targets for future therapies. Our previous work demonstrated that SIRT2 plays a critical role in the repair of cisplatin-induced DNA crosslink lesions through promotion of transcription-coupled NER in DRG neurons. Moreover, SIRT2 overexpression in transgenic *Sirt2-*knockin mice conferred protection against CIPN.^14^

SIRT2, an NAD^+^-dependent deacetylase in the sirtuin family, has been implicated in multiple biological processes including tumor suppression, neurodegenerative disorders, lipid and glucose homeostasis, and longevity.^15–19^ SIRT2 is found in both the nucleus and cytoplasm,^20,21^ and its localization and expression are regulated through diet, oxidative stress,^20,22^ and cell cycle progression.^23^ Pharmacologic activation of SIRT2 by resveratrol^24^ and nicotinamide riboside (NR)^25^ has been shown to alleviate diabetic neuropathy in animal models.^26^

The current study examined the effect of SIRT2 activation by NR supplementation on CIPN in mouse models. We provide genetic evidence that NR supplementation effectively prevents and treats CIPN in a SIRT2-dependent manner. We also present in vitro data demonstrating the specific neuroprotective effects of NAD^+^ on neuronal cells without affecting cytotoxicity of cisplatin in lung and head and neck cancer cell models. Combined with the growing preclinical data supporting NAD^+^ in neuroprotection and the feasibility of NR supplementation, we encourage initiation of a phase I clinical trial to assess the safety of NR in patients with CIPN.

## Materials and Methods

### Cell Culture

Mouse Lewis lung carcinoma cell line LL/2 (LLC), human non-small cell lung cancer cell line H1299, and human tongue squamous cell carcinoma cell line SCC-25 were purchased from ATCC. LLC cells were cultured in Dulbecco’s modified Eagle’s medium (Gibco) with 10% fetal bovine serum (FBS), penicillin (100 µg/mL), and streptomycin (100 µg/mL) (Gibco) and maintained in a 37°C incubator with 5% (v/v) CO_2_. H1299 and SCC-25 cells were cultured in RPMI-1640 media (Gibco) with 10% FBS, penicillin (100 µg/mL), and streptomycin (100 µg/mL) and maintained in a 37°C incubator with 5% (v/v) CO_2_. The 50B11 cell line, immortalized neuronal cells derived from rat DRG sensory neurons, was provided by Ahmet Hoke (Johns Hopkins University). Differentiation and axonal elongation are induced in culture with forskolin, and the majority of cells stop dividing and begin to extend neurites within hours. Cells were maintained in neurobasal medium with 10% FBS, 0.2% glucose, 0.5 mM l-glutamine, and 1% penicillin–streptomycin (Gibco).^27^

### Mouse Strains

C57BL/6 (*Sirt2*-WT) mice were purchased from the NCI (Charles River Lab), and SIRT2^−/−^ C57BL/6 (*Sirt2*-KO) mice were obtained from Tiago F. Outeiro (Department of Neurodegeneration and Restorative Research, Center for Nanoscale Microscopy and Molecular Physiology of the Brain, University Medical Center Göttingen).^28^ The genotypes of all mice were verified by PCR-based genotyping. Six- to eight-week-old male or female mice were used with an equal distribution between sex. All mice were bred and maintained in the Department of Laboratory Animal Medicine (DLAM) at the University of Arkansas for Medical Sciences.

### Cisplatin-Induced Peripheral Neuropathy

Peripheral neuropathy was induced by daily intraperitoneal (i.p.) injections of cisplatin (50 mL vial, Fresenius KABI, suspended in saline) at 2.3 or 5.0 mg/kg for low or high dose, respectively. Two cycles were administered with 5 consecutive daily injections in each cycle and a 5-day rest in between the 2 cisplatin treatment cycles. On days 0, 7, 22, and every 10 days thereafter, electronic von Frey tests were performed to evaluate mechanical allodynia.

### Effect of NR on CIPN

Daily i.p. NR (500 mg/kg) injections were administered to mice starting on day 1 for prevention models. Treatment models administered daily i.p. injections on days 32–62. For mice treated with cisplatin along with NR, 2 cycles of i.p.-injected cisplatin (2.3 or 5.0 mg/kg) were given along with daily NR injections as described in the CIPN model. In vivo SIRT2 expression and activity were verified via western blot of mouse liver samples.

### Tactile Allodynia Assay

Static mechanical pain hypersensitivity was assessed in mice using the electronic von Frey system (Dynamic Plantar Aesthesiometer, Ugo Basile) to measure thresholds of tactile allodynia. The mice were placed in a chamber box with a mesh screen floor, and a single, unbending filament was applied vertically to the mid-plantar region of both hind paws with increasing force (*g*) until a paw-withdrawal response was elicited. The force at which this response occurred was electronically recorded and was designated as the paw-withdrawal threshold (PWT) by the apparatus. These steps were repeated 3 times and the average measurement was calculated and recorded.^29,30^ Relative PWT represents the PWT of cisplatin- and NR-treated mice divided by the PWT of saline- and NR-treated mice, respectively.

### Heat Hypersensitivity Assay (Hot Plate Test)

Heat hypersensitivity was tested using a plantar hot plate analgesia meter, as previously described (IITC Life Science Inc).^31,32^ The mice were individually placed on a hot plate that was maintained at a temperature of 51.0 ± 0.1°C. The latency (seconds) to the first sign of hind paw licking or jumping or a jump response to avoid thermal pain was taken as an index of pain threshold and was monitored using an electronic timer. Decreases in withdrawal latency corresponded to increased sensitivity to heat stimuli (10–12). Results were reported as the mean value of 3 readings.

### SIRT2 KO With CRISPR/Cas9 Gene Editing

Five pairs of SIRT2 single guide RNAs (sgRNAs) designed by the ATUM CRISPR design tools (http://www.atum.bio/catalog/vectors/grna-design) were prepared for screening. The oligos were designed based on the target site sequence (20 bp) and were flanked on the 3′ end by an NGG PAM sequence. Lenti-CRISPR-v2 (Addgene, 52961) contained 2 expression cassettes, hSpCas9 and the chimeric guide RNA. The vector was digested using BsmBI, and a pair of annealed oligos was subcloned into the sgRNA scaffold. Then, the cloned sgRNA lenti-CRISPR-v2 vector was sequenced using the hU6 promoter primer. The lenti-CRISPR-v2 plasmid (with sgRNA cloned) was cotransfected into HEK293T cells with the packaging plasmids pVSVg (Addgene, 8454) and psPAX2 (Addgene, 12260). CMV-EGFP was used as a positive control for viral production. The lentivirus was concentrated by centrifugation at 20 000*g* for 2 hours at 4°C. 50B11 cells were infected by the concentrated lentivirus. They were selected 48 hours later by 2 µg/mL puromycin, incubated for another 48 hours, and then harvested to detect SIRT2 expression by western blotting. sgRNA targeting 5′-GCGGAAGT-CAGGGATTCCTG-3′ showed optimal functionality with rat SIRT2. For controls, an empty vector was transfected to maintain SIRT2 expression.

### Western Blot

Cells were harvested and washed with ice-cold phosphate-buffered saline (PBS). Cells were lysed with RIPA lysis buffer (Boston BioProducts) containing 1× protease and phosphatase inhibitors (Thermo Fisher Scientific). 40 µg of proteins were separated by sodium dodecyl sulfate–polyacrylamide gel electrophoresis (GenScript) and transferred onto PVDF membranes (GE Healthcare). Membranes were incubated in 5% milk for 2 hours for blocking and probed with primary antibodies overnight at 4°C. The next day after washing with TBST, HRP-conjugated secondary antibodies (Cell Signaling Technology) were added for 1 hour at room temperature. After washing, protein bands were detected in UVITEC Alliance with ECL (PerkinElmer Western Lightning Plus-ECL). Western blot results were then analyzed with ImageJ application to quantify the protein levels.

### Cell Survival Assay

The following assays were performed as previously described.^14^ Briefly, 50B11 (*Sirt2*-WT cell line with vector and *Sirt2*-KO obtained via CRISPR/Cas9 gene editing) cells were seeded in 6-well plates. After induction of differentiation for 24 hours,^27^ cells were treated with cisplatin at 1 and 2 µg/mL. H1299 and SCC-25 cells were plated and treated with 1 and 2 µg/mL cisplatin. Cell survival was assessed at 24, 48, and 72 hours after cisplatin treatment. For NAD^+^ treatment, differentiated 50B11, H1299, and SCC-25 cells were pretreated with 5 µM NAD^+^ for 1 hour followed by cisplatin treatment. At the end of the experiments, 6-well plates were kept on ice, and the number of live cells from each sample was determined using an Automated Cell Counter (Bio-Rad, TC20) after trypan blue staining. The survival fraction was calculated as the number of cells that survived after drug treatment normalized to the number of cells that survived after vehicle treatment times 100.

### Immunohistochemistry

After treatment, mice were euthanized and dissected. Fresh mouse DRG samples were isolated and immediately fixed with formalin. Paraffin-embedded DRG tissue sections were prepared, and slides were processed for IHC staining as previously described.^14^ In brief, sections were deparaffinized and rehydrated. They were then pretreated with Target Retrieval Solution, pH 6.0 (DAKO). Sections were blocked with Protein Block (Abcam) and then incubated with primary antibody against SIRT2 (Proteintech), Acetyl-α-Tubulin (Cell Signalling), and anti-NeuN antibody (Abcam). After overnight incubation with primary antibodies, slides were washed and incubated with secondary goat anti-mouse Alexa Fluor 594-conjugated antibodies or goat anti-rabbit Alexa Fluor 488-conjugated antibodies (Thermo Fisher Scientific) and analyzed by fluorescence microscopy (Carl Zeiss). Immunoreactivity was quantified using ImageJ, and expressions from each mouse DRG were plotted.

### Subcutaneous LLC Mouse Model

Six- to eight-week-old C57BL/6 mice were inoculated subcutaneously under anesthesia with 5 × 10^5^ LLC cells suspended in 100 µL ice-cold PBS. Each injection was given in the dorsal flank region. The day of tumor cell implantation was designated as day 0 and tumor size was measured from day 6. Tumors were measured every other day and harvested on day 25. NR was injected i.p. at a dose of 500 mg/kg throughout the experiment. Cisplatin was also injected i.p. at 2.3 mg/kg for 2 cycles, with 5 consecutive daily injections in each cycle and a 5-day rest in between the 2 cisplatin treatment cycles.

### Statistics

Data are presented as mean ± SEM and are representative data from 3 independent experiments, unless otherwise indicated, for all in vitro studies. Statistical analysis of the differences among groups was performed with Prism 6.0 (GraphPad) for Windows using 1-way ANOVA with Tukey post-test, 2-way ANOVA with Bonferroni post-test, or 2-tailed Student’s *t* test. *P < .05 was deemed statistically significant.

### Study Approval

All procedures were approved by the University of Arkansas for Medical Sciences Institutional Animal Care and Use Committee (IACUC). All animal experiments were performed in accordance with NIH regulations on the use and care of experimental animals.

## Results

### NAD^+^ Protects Neuronal Cells From Cisplatin-Induced Cytotoxicity

**Figure 1.**
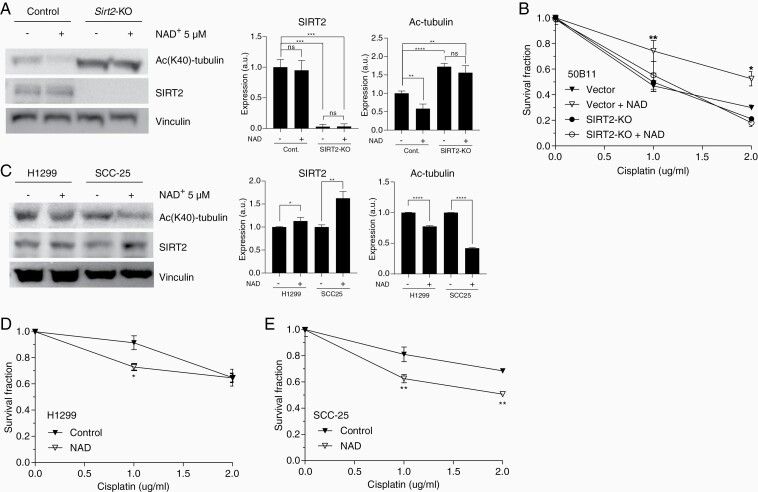
NAD^+^ protects neuronal cells, but not cancer cells, from cisplatin-mediated cytotoxicity. (A) Western blot and quantification showing SIRT2 expression and deacetylase activity in *Sirt2*-KO and control 50B11 cells with and without NAD^+^ supplementation. (B) *Sirt2* KO of differentiated 50B11 neuronal cells resulted in decreased survival when treated with cisplatin for 48 hours in a dose-dependent manner. NAD^+^ supplementation (5 µM) improved survival only in vector control 50B11 cells which express SIRT2. (C) Western blot and quantification showing the effect of NAD^+^ on SIRT2 expression and activity in H1299 and SCC-25 cells. The effect of NAD^+^ on cell survival of human H1299 lung cancer cells (D) and human SCC-25 head and neck squamous cell carcinoma cells (E) after cisplatin treatment for 48 h. *n* = 3. Data points are mean values from 3 repeat experiments ± SEM and were analyzed by 1-way ANOVA with post hoc Tukey test. *P < .05; **P < .01 ; ***P < .001; ****P < .0001.

Peripheral neuron cytotoxicity, particularly in the DRG, is thought to play an integral role in the development of CIPN. We have previously demonstrated that SIRT2-dependent NER plays a critical role in the repair of cisplatin-induced DNA damage in DRG neuronal cells. To investigate whether pharmacological activation of SIRT2 by administration of NAD^+^ protects peripheral neurons from cisplatin-induced cell death, we supplemented neuronal cells with NAD^+^ and assessed cell cytotoxic response to cisplatin treatment. Cell death was measured by positive trypan blue staining in neuronally differentiated 50B11 cells in which SIRT2 expression was manipulated using the CRISPR/Cas9 lentivirus system.^33,34^ Western blot in Figure 1A demonstrates the effect of NAD^+^ on SIRT2 expression and enhanced deacetylase activity measured by the level of acetylation on lysine 40 (AcK40) of α-tubulin, a classic SIRT2 substrate. *Sirt2-*KO and wild-type (WT) cells showed a significant decrease in cell survival when treated with cisplatin (Figure 1B). Supplementation of NAD^+^ had no effect on cisplatin-induced cytotoxicity in *Sirt2*-KO 50B11 cells, however, *Sirt2*-WT cells showed improved survival when NAD^+^ was administered in addition to cisplatin (Figure 1B). Importantly, NAD^+^ supplementation in cancer cells activated SIRT2 (Figure 1C) but did not inhibit cisplatin-induced cytotoxicity against lung (Figure 1D) and tongue cancer cells (Figure 1E). In fact, NAD^+^ sensitized SCC-25, a human head and neck squamous cell carcinoma (SCC) cell line, to cisplatin (Figure 1E). Cell survival assays were repeated with 24- and 72-hour cisplatin treatments with similar results. NAD^+^ improved survival of WT 50B11 cells but not *Sirt2*-KO cells at 72 hours. No difference was observed at 24 hours (Supplementary Figure 1A and B). Cisplatin-induced cytotoxicity of cancer cells lines was not affected by NAD^+^ supplementation (Supplementary Figure 1C–F). These data suggest NAD^+^ protects neuronal cells against cisplatin-induced cytotoxicity without compromising the efficacy of cisplatin treatment on cancer cell killing.

### NR Administration Mitigates CIPN

**Figure 2.**
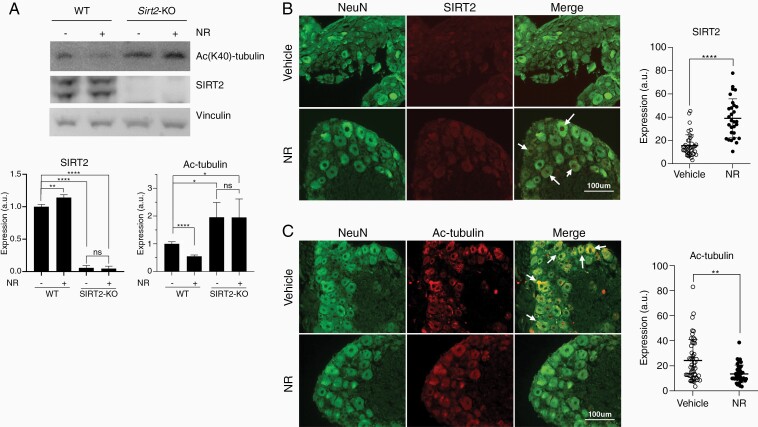
Nicotinamide riboside activates SIRT2. (A) Western blot and quantification show SIRT2 expression and deacetylase activity increase in liver with NR supplementation, as indicated by decreased levels of α-tubulin K40 acetylation (AcK40), in WT but not *Sirt2*-KO C57BL/6 mice (*n* = 3). Representative images (×20 magnification) and quantification of IHC-stained (B) SIRT2 and (C) acetyl-tubulin in WT mouse DRG with and without NR treatment. Arrows indicate (B) SIRT2 and (C) acetyl-tubulin present in mature neurons as visualized by NeuN staining. *n* = 42 and 50, respectively. Statistical significance was analyzed by 2-tailed Student’s *t* test. *P < .05; **P < .01; ****P < .0001. DRG, dorsal root ganglia; NR, nicotinamide riboside; WT, wild-type.

**Figure 3.**
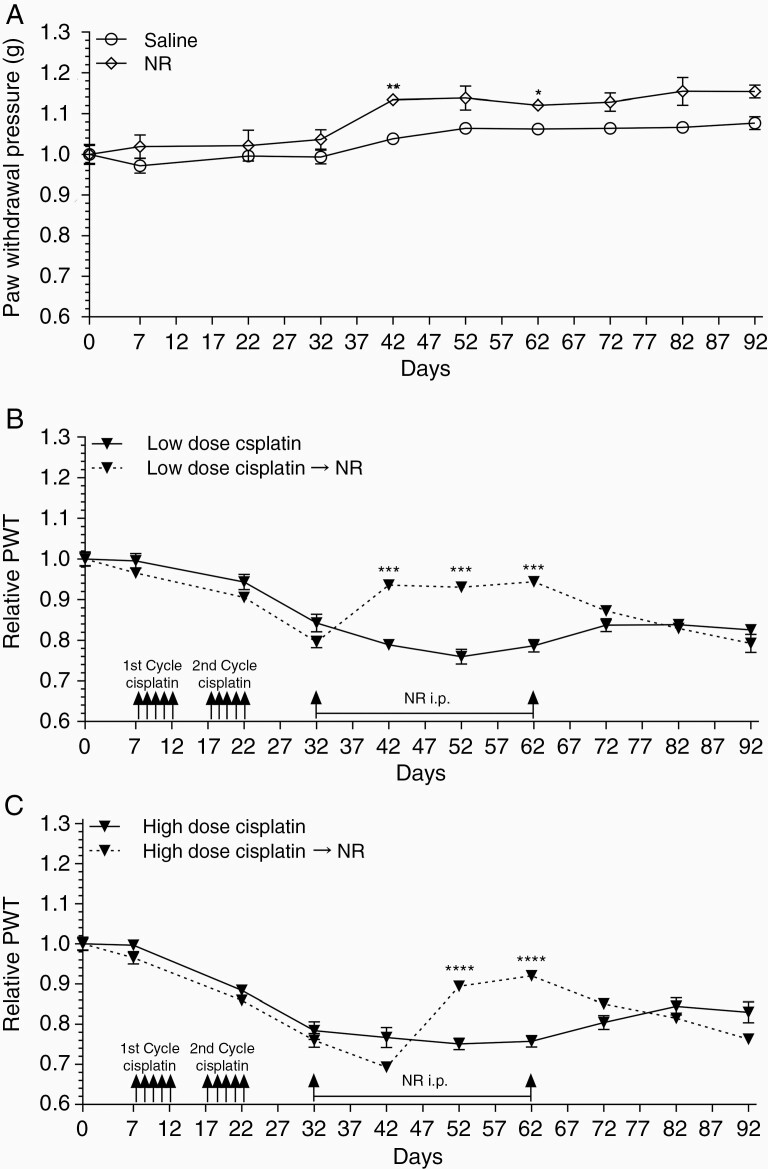
Nicotinamide riboside reverses CIPN. (A) Mechanical sensitivity of WT mice as measured by von Frey tests while receiving daily i.p. saline or NR (500 mg/kg) injections (*n* = 5). Response of WT mice before and after (B) low- (2.3 mg/kg, *n* = 6) and (C) high-dose (5.0 mg/kg, *n* = 8) cisplatin treatment. Relative paw-withdrawal threshold (PWT) is the PWT of mice treated with cisplatin ± daily NR normalized to saline or NR control groups, respectively. Data points are mean values ± SEM and were analyzed by 2-way ANOVA analysis with post hoc Bonferroni test. *P < .05; **P < .01; ***P < .001; ****P < .0001. CIPN, cisplatin-induced peripheral neuropathy; NR, nicotinamide riboside; WT, wild-type.

While our genetic mouse model has illustrated SIRT2 overexpression protects against CIPN,^14^ pharmacologic activation of SIRT2 is required for clinical application. We first investigated whether pharmacologic activation of SIRT2 by NR, the NAD^+^ precursor, can reverse CIPN in a mouse model. Western blot demonstrates SIRT2 activation by its increased deacetylase activity with NR supplementation in the liver from WT, but not *Sirt2*-KO, mice (Figure 2A). The effect of NR on SIRT2 activity in DRG was assessed via immunohistochemical staining of DRG harvested from WT mice treated with daily NR (500 mg/kg) for 5 days. An increase in SIRT2 expression and activity, as demonstrated by decreased acetyl-tubulin, was observed with NR supplementation compared to vehicle (Figure 2B and C). CIPN was induced in WT C57BL/6 mice by daily i.p. injection of cisplatin at low (2.3 mg/kg) or high (5.0 mg/kg) dose for 5 days beginning on day 8. The first cycle was followed by 5 days of rest and then an additional 5 days of cisplatin. The experimental group underwent cisplatin treatment followed by daily i.p. injections of NR (500 mg/kg) days 32 through 62. Because CIPN commonly manifests in patients as hyperalgesia, an increased perception of pain, we assessed mechanical thresholds (ie, paw-withdrawal pressure in grams) using von Frey filaments before and after cisplatin administration. Mechanical thresholds were tested on days 0, 7, 22, and every 10 days thereafter through day 92. Mice receiving saline injections showed relatively stable mechanical thresholds throughout treatment. Comparatively, those receiving daily NR (500 mg/kg) showed an elevated mechanical threshold on day 42 that became nonsignificant as the experiment continued (Figure 3A). Conversely, mice receiving low-dose cisplatin developed CIPN as demonstrated by significant decreases in mechanical threshold relative to their respective saline or NR controls at day 22 and through the end of the treatment course (Figure 3B). Importantly, administration of NR 10 days after 2 cycles of cisplatin treatment reversed CIPN in mice as demonstrated by a restoration of mechanical threshold measured on day 42 of cisplatin and day 10 of daily NR administration. This therapeutic effect was sustained during the daily NR injections. Intriguingly, NR-mediated alleviation of CIPN dissipated once NR was discontinued (Figure 3B). We also examined if NR could mitigate CIPN from high-dose cisplatin. We observed decreased thresholds compared to saline-treated mice, but with a greater magnitude compared to low dose. NR treatment again restored thresholds starting at day 52, approximately 10 days slower than in the low-dose cisplatin-treated group (Figure 3C). Similarly, discontinuation of NR after 30 days of treatment led to recurrence of CIPN when measured at day 82 (Figure 3C).

This effect was validated using dynamic hot plates to assess thermal thresholds (ie, paw-withdrawal latency in seconds). CIPN was induced using 2 5-day cycles of cisplatin (2.3 mg/kg) as depicted by the schematic (Supplementary Figure 2A). Thermal thresholds were tested on days 0, 15, 25, 35, 45, and 55. Cisplatin-treated mice developed CIPN as demonstrated by decreased thermal thresholds on days 15 and 25. Treatment with daily NR (500 mg/kg) restored thermal thresholds to levels equivalent to saline-treated mice. A small increase in thermal thresholds was observed in all groups and may represent development of hot plate tolerance following repeat stimulation and subsequent keratinization of mouse hind paws (Supplementary Figure 2B).

These data suggest that pharmacologic activation of SIRT2 reverses CIPN and likely requires continued treatment to maintain the therapeutic effect.

### Prophylactic and Concurrent NR Treatment Protects Mice From CIPN

**Figure 4.**
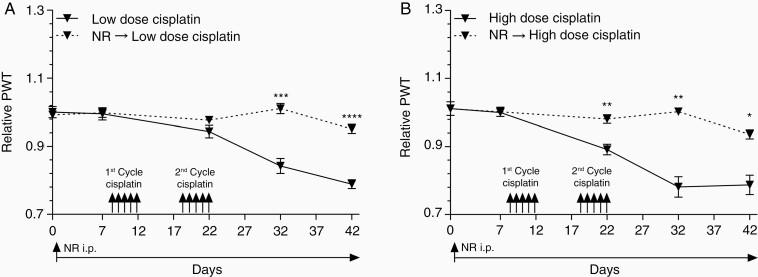
Nicotinamide riboside prevents CIPN. Mechanical allodynia measured by von Frey tests before and after (A) low- (2.3 mg/kg) and (B) high-dose (5.0 mg/kg) cisplatin treatment. Daily NR supplement (500 mg/kg) was started prior to cisplatin treatment on day 1. Mechanical sensitivity was compared between mice treated with cisplatin and NR followed by cisplatin. Relative PWT is the PWT of mice treated with cisplatin or NR followed by cisplatin normalized to saline and NR groups. *n* = 6. Data points are mean values ± SEM and were analyzed by 2-way ANOVA analysis with post hoc Bonferroni test. *P < .05; **P < .01; ***P < .001; ****P < .0001. CIPN, cisplatin-induced peripheral neuropathy; NR, nicotinamide riboside; PWT, paw-withdrawal threshold.

In addition to the treatment model, we evaluated whether pharmacologic SIRT2 activation by NR before exposure to cisplatin could serve as a strategy for CIPN prevention. The dual-dose CIPN model was utilized as described above, and mechanical thresholds were measured on days 0, 7, 22, 32, and 42. NR-treated mice began receiving daily i.p. injections of NR at 500 mg/kg on day 1 (7 days before first dose of cisplatin). Based on our observation that CIPN recurs upon discontinuation of NR (Figure 3B and C), we extended NR supplementation during and following cisplatin treatments. As expected, mice receiving low-dose cisplatin developed CIPN as demonstrated by reduced mechanical thresholds relative to saline-treated mice (Figure 4A). Interestingly, prophylactic and concurrent administration of NR prevented mice from developing CIPN despite receiving cisplatin (Figure 4A).

Again, high-dose cisplatin induced more severe CIPN as illustrated by a larger decrease in relative mechanical threshold compared to that caused by low-dose cisplatin (Figure 4B). Despite cisplatin’s observed dose-dependent toxicity, prophylactic and concurrent NR administration still effectively prevented CIPN development in mice receiving high-dose cisplatin.

Assessment of CIPN using thermal thresholds demonstrated similar results. Mice were treated with cisplatin as illustrated in the treatment schematic (Supplementary Figure 3A), and thermal thresholds were measured approximately every 10 days. Mice treated with cisplatin alone developed progressive CIPN as illustrated by reduced thermal thresholds compared to saline-treated mice. Interestingly, administration of daily NR (500 mg/kg) prior to, during, and following cisplatin treatment prevented the development of CIPN as seen by stable thermal thresholds compared to baseline and saline-treated mice (Supplementary Figure 3B).

### Prophylactic and Concurrent NR Treatment Does Not Affect In Vivo Tumor Response to Cisplatin

**Figure 5.**
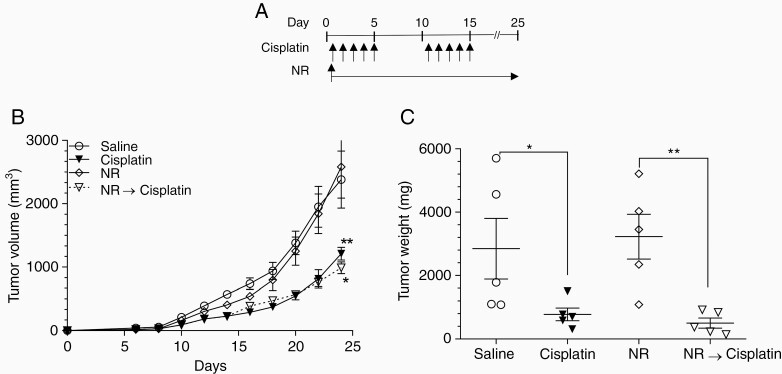
Nicotinamide riboside does not inhibit cisplatin toxicity in carcinoma model. (A) C57BL/6 mice bearing LLC tumors were treated with 2 cycles of cisplatin or saline in the presence or absence of NR. (B) Tumor volume was monitored for 25 days at which time tumors were harvested and (C) weighed. *n* = 5. Data points are mean values ± SEM and were analyzed by 2-way ANOVA analysis with post hoc Bonferroni test and 1-way ANOVA with post hoc Tukey test. *P < .05; **P < .01. LLC, Lewis lung carcinoma; NR, nicotinamide riboside.

To investigate whether NR supplementation affects cisplatin-mediated tumor control, we examined allograft LLC progression in WT mice. Mice were subcutaneously inoculated with LLC cells, and tumor growth was monitored for 25 days in the presence and absence of cisplatin before harvesting tumors. Cisplatin (2.3 mg/kg) was given daily via i.p. injection on days 1–5 and 11–15 (Figure 5A). Daily NR (500 mg/kg) was given throughout the experiment but did not affect LLC response to cisplatin. Mice receiving cisplatin showed significantly reduced tumor growth compared to mice not treated with cisplatin regardless of NR treatment (Figure 5B and C). Together these data suggest NR prevents and treats CIPN without hindering cisplatin-mediated tumor control.

### NR Requires SIRT2 for CIPN Prevention and Treatment

**Figure 6.**
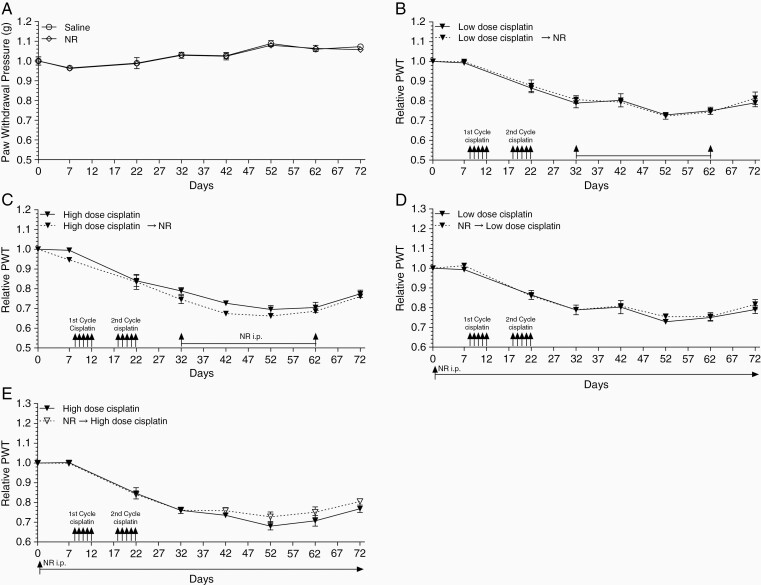
CIPN treatment and prevention by NR is dependent on SIRT2. (A) Response of saline- and NR-treated *Sirt2*-KO mice to mechanical stimulation (*n* = 5). Mechanical sensitivity of *Sirt2*-KO mice before and after (B) low- (2.3 mg/kg) and (C) high-dose (5.0 mg/kg) cisplatin treatment. CIPN treatment was investigated by daily NR (500 mg/kg) treatment starting 10 days after the end of cisplatin treatment. NR treatment was continued for 30 days. *n* = 6 in cisplatin alone groups, *n* = 4 in low-dose cisplatin followed by NR, and *n* = 5 in high-dose cisplatin followed by NR. Relative PWT of *Sirt2*-KO mice as a measure of mechanical sensitivity before and after (D) low- (2.3 mg/kg) and (E) high-dose (5.0 mg/kg) cisplatin treatment. NR-treated mice received daily NR (500 mg/kg) injections starting day 1. *n* = 6 in cisplatin alone groups and *n* = 4 in NR prevention groups. Data points are mean values ± SEM and were analyzed by 2-way ANOVA analysis with post hoc Bonferroni test. CIPN, cisplatin-induced peripheral neuropathy; NR, nicotinamide riboside; PWT, paw-withdrawal threshold.

Our previous work identified SIRT2 as a mediator of NER-dependent protection against CIPN in a genetic mice model.^14^ We have shown that NR, the pharmacologic activator of SIRT2, exhibits significant protective effect as shown in the CIPN treatment and prevention models described above. NR is also known to activate other SIRT family members, such as SIRT1. We have previously shown, however, that exposure of cultured neuronal cells to NAD^+^ enhances the efficiency of transcription-coupled NER and subsequent resolution of cisplatin-induced DNA crosslinks in a SIRT2-, but not SIRT1-dependent manner.^14^ To examine whether NR-mediated CIPN protection in mouse is dependent on SIRT2, we investigated whether NR supplementation could protect *Sirt2*-KO mice from CIPN using the treatment model described above. Western blot demonstrates increased SIRT2 expression and deacetylase activity with NR supplementation of WT, but not *Sirt2*-KO, mice (Figure 2A). *Sirt2*-KO mice receiving saline or NR injections maintained stable mechanical thresholds throughout the experiment (Figure 6A). Mice receiving cisplatin developed dose-dependent CIPN beginning after the second cisplatin cycle as illustrated by a significant reduction in mechanical threshold relative to saline and NR control groups with a greater decrease seen in mice receiving high-dose cisplatin. Interestingly, treatment with NR following onset of CIPN did not return mechanical thresholds to those shown in saline- or NR-treated mice. In fact, there was no difference in mechanical threshold between cisplatin-treated mice and mice receiving NR in addition to cisplatin treatment (Figure 6B and C).

Similarly, lack of preventive effect of CIPN with prophylactic and concurrent treatment of NR in *Sirt2*-KO mice was observed in the prevention model. Low- and high-dose cisplatin-induced CIPN in a dose-dependent manner that continued through the end of the experiment. Importantly, NR supplementation prior and concurrent to cisplatin treatment showed no protective effect against CIPN development even when continued through day 72 (Figure 6D and E). Assessment of CIPN with dynamic hot plates showed the same dependence on SIRT2. Cisplatin and NR were administered as shown (Supplementary Figure 4A). *Sirt2-*KO mice treated with cisplatin developed reduced thermal thresholds compared to saline- and NR-treated mice regardless of prophylactic and concurrent NR administration (Supplementary Figure 4B). Together, these data demonstrate a dependence on SIRT2 for NR-mediated CIPN prevention and treatment.

## Discussion

NR, a naturally occurring NAD^+^ precursor, is garnering much attention due to recently discovered implications in cardiovascular, endocrine, and neurodegenerative diseases as well as longevity.^35^ Many of these beneficial effects are thought to be mediated by sirtuins, the NAD^+^-dependent deacetylases.^35^ Here, we demonstrate that NR supplementation prevents mice from developing CIPN and reverses CIPN if already established. We provide genetic evidence that NR-mediated CIPN prevention and treatment is dependent upon SIRT2, a member of the sirtuin family. Finally, we provide data that show pharmacological activation of SIRT2 protects neuronal cells against cisplatin-induced cytotoxicity without compromising cancer cell sensitivity.

NAD^+^ has been implicated in a wide variety of molecular pathways, and its mechanism in peripheral neuropathy is not well understood. We previously identified NER-dependent SIRT2-mediated protection against CIPN,^14^ however, we acknowledge that NR activation is not specific to SIRT2. A sirtuin activator, resveratrol is also available, although it too lacks specificity.^36^ Our data suggest NR is dependent on SIRT2 for CIPN protection (Figure 6), however, use of a more specific SIRT2 activator would be of great clinical benefit to avoid nonspecific effects.

Because NAD^+^ plays a role in diverse metabolic pathways,^37,38^ it serves as a potential target for numerous pathophysiological conditions^39,40^ which could be achieved through NR treatment. In fact, NR supplementation has been shown to prevent axonal degeneration^41^ and neuroinflammation^42,43^ in mice. NR’s neuroprotective effects also have implications in Alzheimer’s disease, Parkinson’s disease, and neuromuscular diseases,^44^ and are primarily mediated by sirtuins.^45^ NR has also been proposed as a strategy to promote longevity by reproducing the NAD^+^ elevation thought to mediate the life-prolonging effects of calorie restriction.^46^ Pharmacokinetic studies of NR have been explored in both human and mouse models and have shown that single oral doses of NR can produce dose-dependent increases in blood NAD^+^ concentration,^25^ further supporting its clinical utility.

Our in vivo model revealed mice treated with NR developed increased mechanical thresholds compared to saline-treated mice on day 42 (Figure 3A). This observation may be the result of NR neuroprotection as described above. Moreover, mice develop hyperkeratosis of their paws as they age which could manifest as falsely elevated mechanical thresholds on von Frey tests. The increased threshold could also indicate a general increase in tolerance to mechanical pressure. Together, the change in skin character and a physiologic tolerance to mechanical pressure could exacerbate the mild effect of NR-induced SIRT2 activation on neuronal health. Given the long history of NAD^+^-mediated longevity,^35^ this observation could indicate that NR provides general health benefits and may maintain nerve health independent of cisplatin injury. NR may provide increased protection from cancer treatment toxicity in select patients or clinical settings, and NAD^+^ expression could act as a predictive marker for toxicity risk and chemotherapy response. Combining better patient selection with an effective therapeutic option could better predict treatment safety and thereby increase the therapeutic index of cisplatin.

While treatment strategies for CIPN are greatly needed, potential drugs must achieve CIPN relief without interfering with the antineoplastic effects of chemotherapy. There has been concern that NAD^+^ supplementation could promote tumorigenesis if NAD^+^ acts as a substrate for actively proliferating cells.^47^ On the contrary, our in vitro data illustrate that NAD^+^ protects neuronal cells from cisplatin-mediated cytotoxicity without inhibiting cisplatin’s antineoplastic effects. In fact, the SCC cell line demonstrated increased sensitivity to cisplatin (Figure 1E). Moreover, our subcutaneous LLC mouse model provides in vivo data demonstrating no difference in cisplatin-mediated tumor control (Figure 5). This is consistent with preclinical data demonstrating antineoplastic effects of NAD^+^ supplementation in lung, liver, and bladder models.^48–50^ Moreover, a phase III trial investigating nicotinamide in nonmelanoma skin cancer prevention showed patients receiving twice daily nicotinamide had a 23% rate reduction of new nonmelanoma skin cancer development compared to placebo.^51^ More data might be available soon as multiple ongoing clinical trials are investigating NAD^+^ precursors as cancer treatment strategies (ClinicalTrials: NCT02416739, NCT04281420, NCT02702492, NCT04677049). The different response to NAD^+^ observed in the 2 cancer cell lines could be due to heterogeneities in the NAD^+^ pathways, as differences in nicotinamide phosphoribosyltransferase (NAMPT), the rate-limiting enzyme in NAD^+^ biosynthesis, allows for selective killing in gastric cancer cells without harming normal tissues.^52^ Further characterization of NAD^+^ metabolism is needed to shed light on the various effects in cancer by NAD^+^.

This study utilizes neuronally differentiated 50B11 cells to investigate whether NAD^+^ protects against cisplatin-induced cytotoxicity and showed that NAD^+^ improves neuronal cell survival in a SIRT2-dependent manner. We have previously illustrated that SIRT2 protects against cisplatin-induced cytotoxicity in primary DRG neurons, 50B11 cells, and differentiated PC12 cells, a common cell model for peripheral neurons. Interestingly, mechanistic studies examining NAD^+^ treatment as a strategy to prevent oxidative cell death have also shown NAD^+^ increases the antioxidant capacity of PC12 cells as mediated by SIRT2.^53^ Activation of SIRT2 by NAD^+^ would likely protect against cisplatin-induced cytotoxicity in these other neuronal cell models.

Promising evidence has shown NR relieves paclitaxel-induced peripheral neuropathy in rats^54,55^ and has led to an ongoing phase II clinical trial (ClinicalTrials: NCT03642990). Although the underlying mechanisms of peripheral neuropathy induced by cisplatin and paclitaxel have not been fully elucidated, the primary mechanisms are distinctly different between the 2 chemotherapy drugs. Cisplatin exerts its antineoplastic effects through formation of DNA platination products, and we have previously demonstrated that enhancement of NER by SIRT2 protects mice against CIPN.^14^ In contrast, paclitaxel induces axonal degeneration through mitochondrial dysfunction, activates macrophages in the DRG resulting in inflammation, and alters Ca^2+^ signaling.^54^ Further investigation of NR for the prevention and treatment of CIPN is warranted.

In conclusion, accumulated evidence demonstrates pharmacological SIRT2 activation with NR effectively protects differentiated DRG neurons, but not proliferating cancer cells, from DNA damage-induced cytotoxicity from cisplatin. This further supports SIRT2-mediated DNA repair pathway as a promising target for pharmacological intervention, as interference with this pathway in platinum-based cancer treatment may enhance the therapeutic index by providing protection of peripheral neuronal function without impeding tumor control.

## Supplementary Material

vdac101_suppl_Supplementary_Figures
