# Supplementary material for: Nicotinamide riboside alleviates cisplatin-induced peripheral neuropathy via SIRT2 activation
Source: Neurooncol Adv. 2022 Jun 27;4(1):vdac101. doi: 10.1093/noajnl/vdac101 (PMC9297957; doi:10.1093/noajnl/vdac101)

*Supplemental Figure Captions*

**Supplemental Figure 1. NAD^+^ protects neuronal cells, but not cancer cells, from cisplatin-mediated cytotoxicity at various time points**

**(A**) *Sirt2* KO of differentiated 50B11 neuronal cells did not affect survival when treated with cisplatin for 24 h. **(B)** *Sirt2* KO decreased survival of 50B11 cells when treated with cisplatin for 72 h in a dose-dependent manner. NAD^+^ supplementation (5 µM) improved survival only in vector control 50B11 cells which express SIRT2. **(C**) The effect of NAD^+^ on cell survival of human H1299 lung cancer cells after **(C)** 24 and **(D)** 72-hour cisplatin treatment. **(E)** The effect of NAD^+^ on cell survival of SCC-25 head and neck squamous cell carcinoma cells after cisplatin treatment for **(E)** 24 and **(F)** 72 h. n = 3. Data points are mean values from three repeat experiments± SEM and were analyzed by one-way ANOVA with post-hoc Tukey test. **P* < 0.05 and ***P* < 0.01.


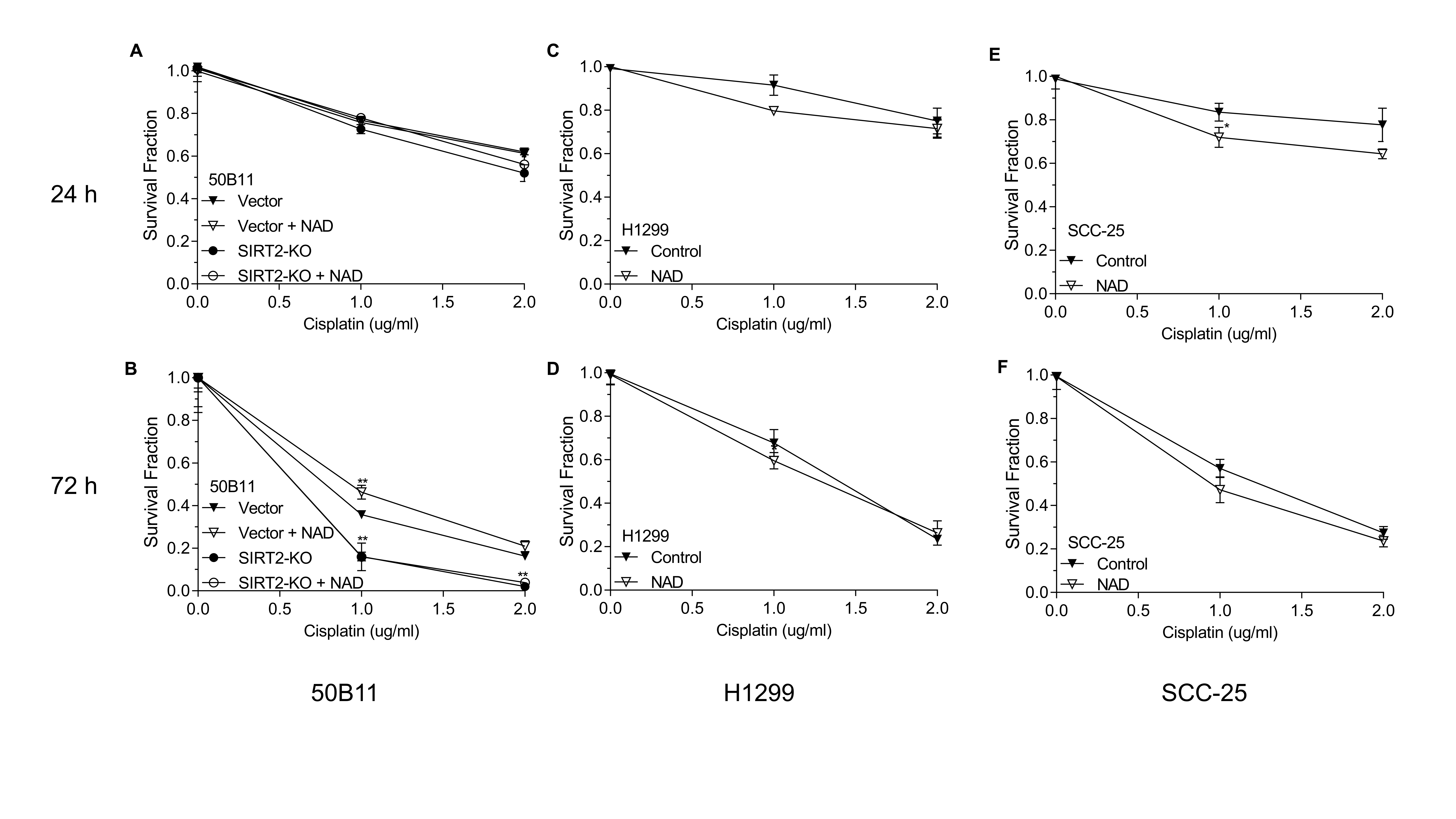


**Supplemental Figure 2. Nicotinamide riboside treats CIPN**

**(A)** Treatment model to induce and treat CIPN with NR in C57BL/6 mice. **(B)** Thermal sensitivity of WT mice as measured by dynamic hot plate tests. Response of mice before and after cisplatin (2.3 mg/kg) treatment in the presence and absence of NR treatment (500 mg/kg). n = 9. Data points are mean values from three repeat experiments± SEM and were analyzed by one-way ANOVA with post-hoc Tukey test. **P* < 0.05; ***P* < 0.01; ****P* < 0.001.


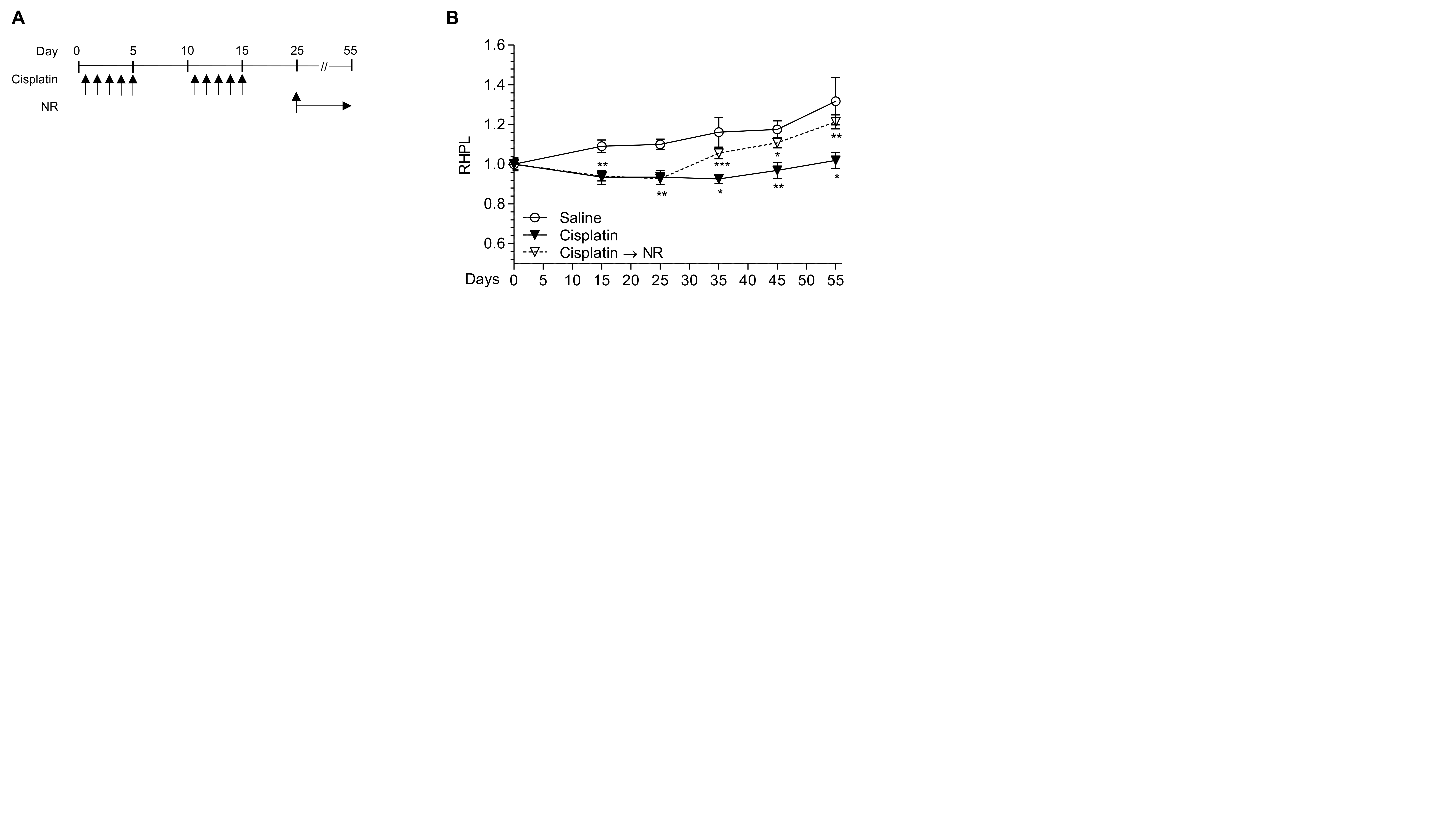


**Supplemental Figure 3. Nicotinamide riboside prevents CIPN**

1. Prevention model using prophylactic and concurrent NR in C56BL/6 mice. **(B)** Thermal sensitivity of WT mice before and after cisplatin (2.3 mg/kg) treatment in the presence and absence of prophylactic and concurrent NR (500 mg/kg). n = 7. Data points are mean values from three repeat experiments± SEM and were analyzed by one-way ANOVA with post-hoc Tukey test. **P* < 0.05 and ***P* < 0.01.


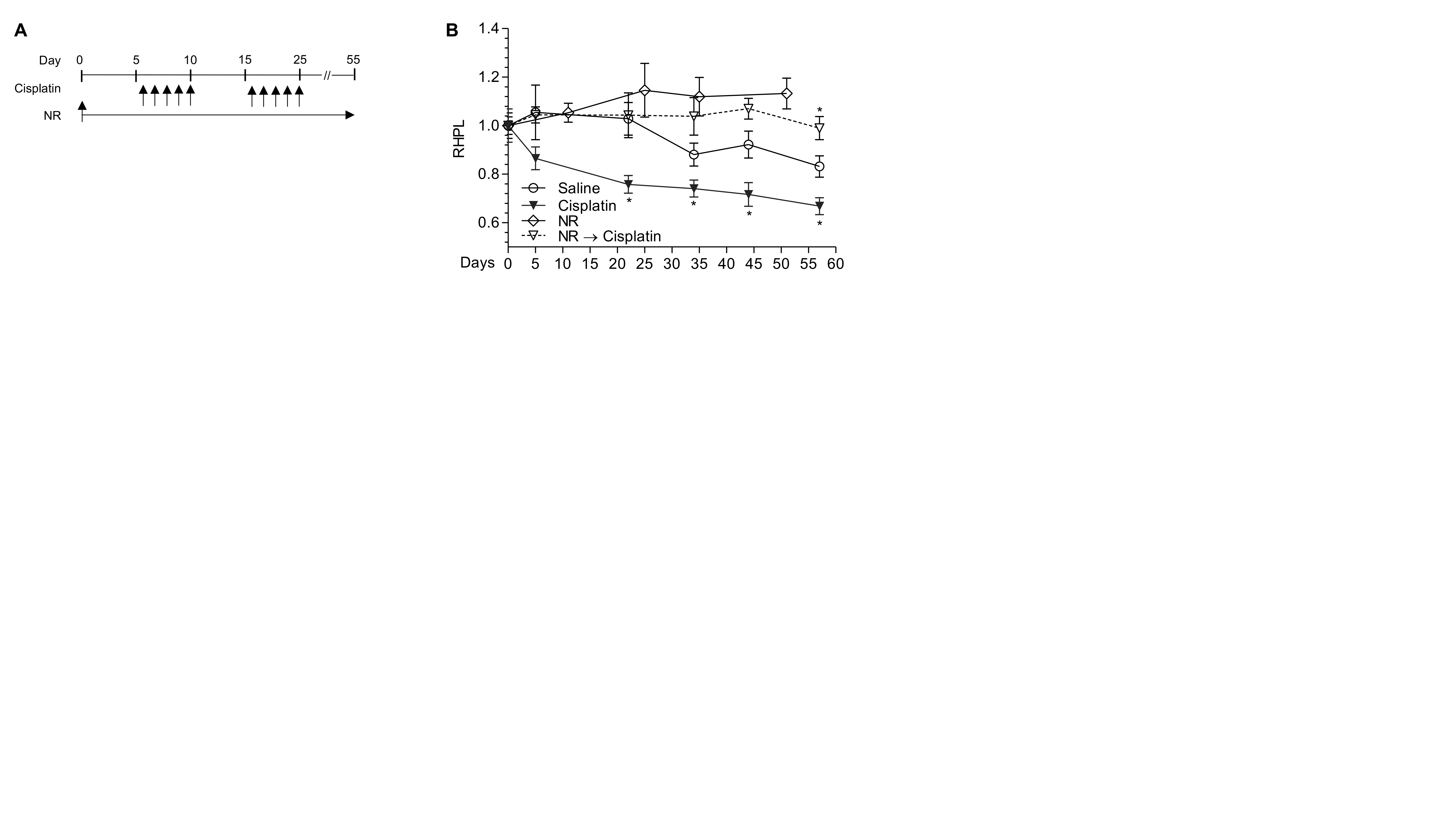


**Supplemental Figure 4. Prevention of CIPN by NR is dependent on SIRT2**

**(A)** Model to prevent the development of CIPN with NR in C57BL/6 mice. **(B)** Thermal sensitivity of *Sirt2*-KO mice before and after cisplatin (2.3 mg/kg) treatment in the presence and absence of prophylactic and concurrent NR (500 mg/kg). n = 7. Relative hot plate latency (RHPL) is the HPL of mice treated with cisplatin ± daily NR normalized to baseline. Data points are mean values ± SEM and were analyzed by two-way ANOVA analysis with post-hoc Bonferroni test.


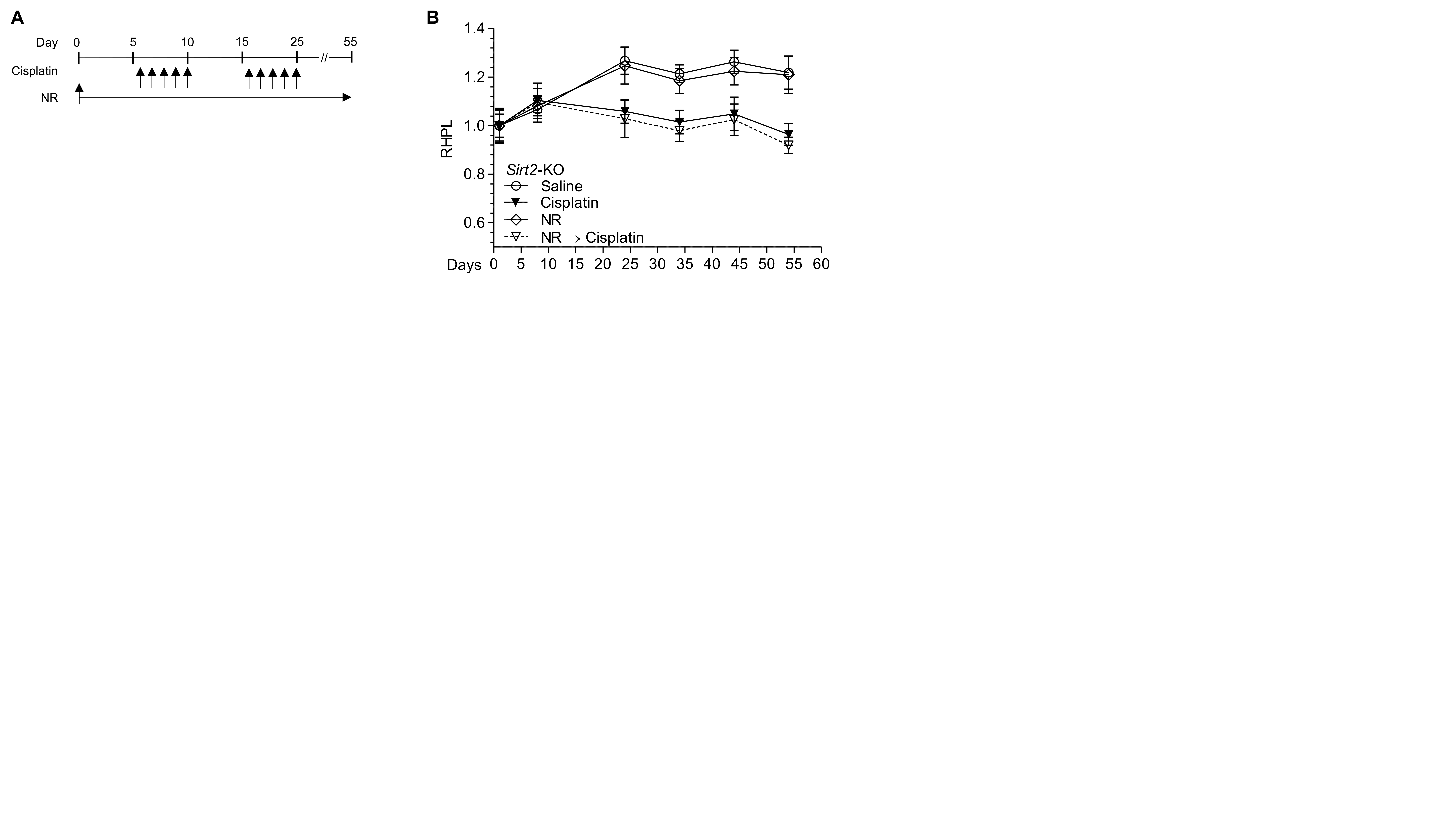

Supplement: vdac101_suppl_Supplementary_Figures [file vdac101_suppl_supplementary_figures.docx]
